# A Giant Lumbar Pseudomeningocele in a Patient with Neurofibromatosis Type 1: A Case Report

**DOI:** 10.1155/2017/4681526

**Published:** 2017-01-31

**Authors:** Mauro Dobran, Maurizio Iacoangeli, Paolo Ruscelli, Martina Della Costanza, Davide Nasi, Massimo Scerrati

**Affiliations:** ^1^Department of Neurosurgery, Università Politecnica delle Marche, Ospedali Riuniti Umberto I, Ancona, Italy; ^2^Department of Emergency Surgery, Ospedali Riuniti Umberto I, Ancona, Italy

## Abstract

This is a rare case of giant lumbar pseudomeningocele with intra-abdominal extension in patient with neurofibromatosis type 1 (NF1). The patient's clinical course is retrospectively reviewed. A 34-year-old female affected by NF1 was referred to our institution for persistent low back pain and MRI diagnosis of pseudomeningocele located at L3 level with paravertebral extension. From the first surgical procedure by a posterior approach until the relapse of the pseudomeningocele documented by MRI, the patient underwent two subsequent posterior surgical procedures to repair the dural sac defect with fat graft and fibrin glue. One month after the third operation, the abdominal MRI showed a giant intra-abdominal pseudomeningocele causing compression of visceral structures. The patient was asymptomatic. The pseudomeningocele was treated with an anterior abdominal approach and the use of the acellular dermal matrix (ADM) sutured directly on the dural defect on the anterolateral wall of the spinal canal. After six months of follow-up the MRI showed no relapse of the pseudomeningocele. Our case highlights the possible use of ADM as an effective and safe alternative to the traditional fat graft to repair challenging and large dural defects.

## 1. Introduction

Pseudomeningoceles are extradural collections of cerebrospinal fluid (CSF) with no dural covering resulting from a dura-arachnoid defect or improper closure during spinal surgery [1]. There are three groups of pseudomeningocele: congenital, traumatic, and iatrogenic. Majority of pseudomeningoceles occurs after lumbar spine surgery and resolves spontaneously. Usually pseudomeningoceles remain embedded in the posterior paraspinal soft tissue and cause no symptoms. The MRI is the radiological examination of choice for diagnosis and follow-up after treatment [2]. Surgical exploration and repair should be reserved for symptomatic cases presenting with clinical features of intracranial hypotension, CSF leak, or infection. Besides pseudomeningocele is an extremely rare cause of spinal cord compression [3]. The association with congenital giant spinal pseudomenigocele and meurofibromatosis type 1 is rare and at the best of our knowledge there is no literature about this association. We describe a case of recurrent spontaneous lumbar giant pseudomeningocele [4] in patient with neurofibromatosis type 1 and the surgical technique to repair the defect.

## 2. Case Presentation

**Figure 1:**
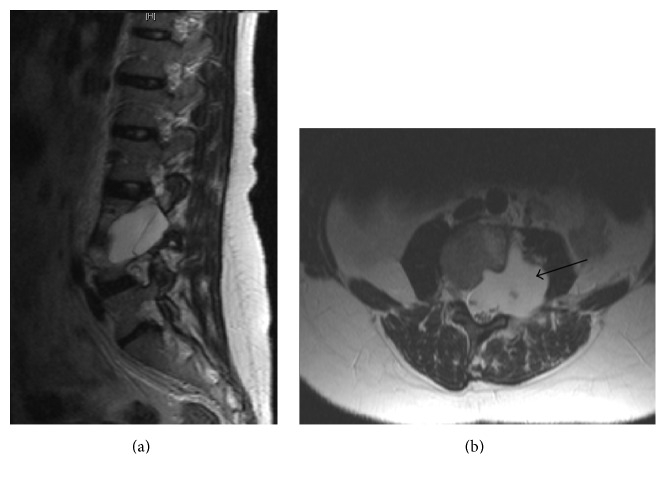
Preoperative MRI sagittal and axial T2-weighted postgadolinium images showed a pseudomeningocele at L3-L4 levels with paravertebral extension and psoas muscle remodeling (black arrow).

**Figure 2:**
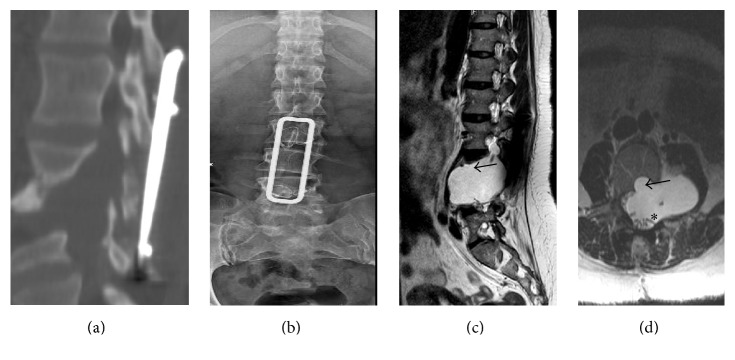
Postoperative CT and X-rays (a and b) showing L3 hemilaminectomy and L2–L5 stabilization with Hartshill rectangle and titanium wire. One-year follow-up after the first operation, sagittal and axial T2-weighted MRI images (c and d) disclosed a relapse of the operated pseudomeningocele at L3-L4 level with vertebral body remodelling (black arrow) and spinal roots displacement and convolution (asterisk).

**Figure 3:**
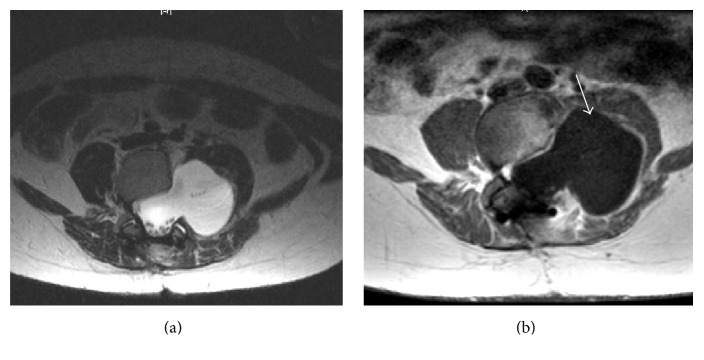
One year after the second operation an axial T2 and T1-weighted postgadolinium MRI images disclosed a pseudomeningocele with roots compression and displacement of the psoas muscle (white arrow).

**Figure 4:**
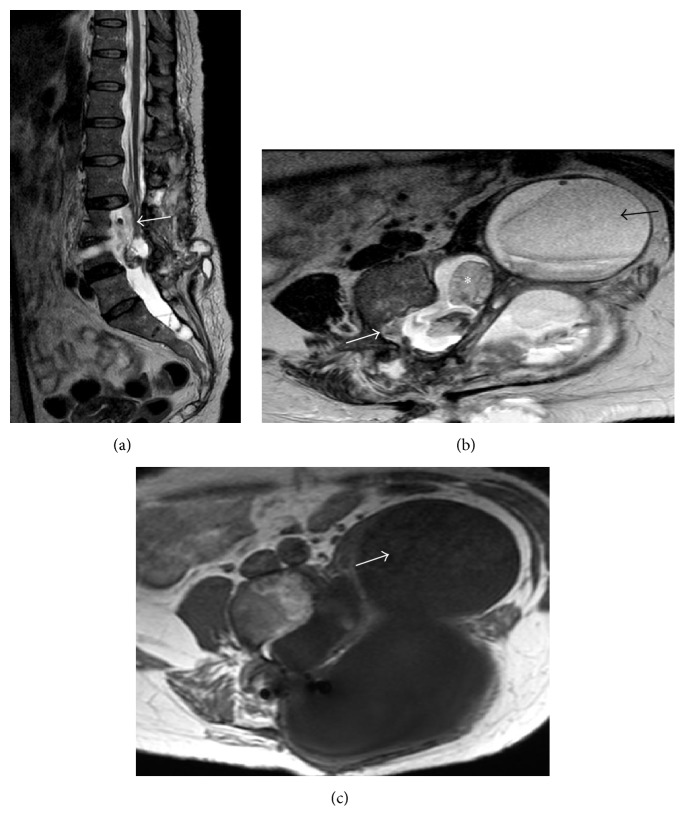
Sagittal and axial T2 weighted (a and b) and axial T1 weighted postgadolinium MRI images at six months after the third operation showed a new giant pseudomeningocele with intra-abdominal extension. The sagittal and axial T2 weighted MRI images (a and b) demonstrated dural sac and cauda roots compression (white arrows) and the inhomogeneous non-CSF like content of the pseudomeningocele (black arrow) probably due to the presence of surgical patch materials and blood products. Axial T1 weighted after gadolinium MRI picture (c) excluded pathological enhancement (white arrow).

**Figure 5:**
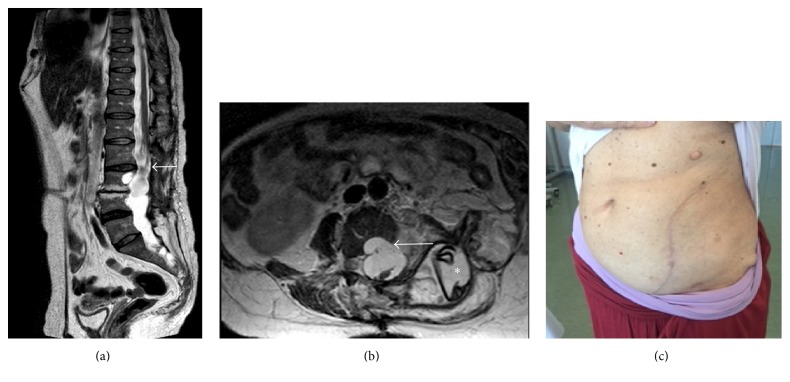
After six months, the patient was fine without neurological deficit or abdominal symptoms and the spinal sagittal and axial T2-weighted MRI images showed no relapse of the pseudomeningocele (a and b). The sagittal T2-weighted MRI demonstrated the decompression of cauda roots (white arrows), even if appeared clumped together due to postsurgical scar. In the axial T2-weighted MRI the residual meningocele (white arrow) and the ADM (asterisk) with fat patch were demonstrated. The surgical incision completely healed was shown in image (c).

This is the case of 34-year-old female with neurofibromatosis type 1 and scoliosis who underwent surgery for low back pain and MRI diagnosis of pseudomeningocele at L3 level with paravertebral extension (Figures 1(a) and 1(b)). The neurological examination was negative. The patient was operated by L3 hemilaminectomy, reconstruction of the dural sac with water tight stitch, and L2–L5 stabilization with Hartshill rectangle and titanium wire (Figures 2(a) and 2(b)) as reported in the treatment of thoracolumbar instability and deformities [5, 6]. At the end of surgery, there was no intraoperative evidence of any residue. After one-year follow-up, the patient referred progressive pain and numbness in L5 left distribution and MRI disclosed a relapse of the operated pseudomeningocele at L3-L4 level (Figures 2(c) and 2(d)). However, since a postoperative MRI was not available, the symptomatic recurrence might be also due to the increase of a hidden residual. A second operation was performed by removing the Hartshill rectangle with posterior reconstruction of the dural sac with fascia lata, fibrin glue, and tight water closure of the dura mater. A postoperative MRI was not available for this time point. After one year an MRI disclosed a pseudomeningocele relapse (Figures 3(a) and 3(b)) and the patient underwent a third surgical procedure by L2–L4 hemilaminectomy and reconstruction by synthetic dura mater and autologous fat grafts with postoperative subarachnoid drainage for one week. After six months MRI showed a giant pseudomeningocele with intra-abdominal extension (Figures 4(a), 4(b), and 4(c)). At the end of each operation, there was no intraoperative evidence of any residual of the pseudomeningocele. However, since postoperative MRI images of the dural sac are not available until the fourth and last surgery, the multiple relapses might be also due to the progression of an unknown residual. The patient was asymptomatic without neurological deficit or abdominal symptoms. The operation was performed via anterior intra-abdominal exposure and reconstruction of the lateral wall of dural sac with a ADM sutured directly on the dural defect with reabsorptive stiches. After six months, the patient was fine without neurological deficit or abdominal symptoms and the spinal MRI showed no relapse of the pseudomeningocele (Figures 5(a), 5(b), and 5(c)).

## 3. Discussion

Neurofibromatosis type 1 (NF1) is a genetic multisystemic disorder involving skin, central and peripheral nervous systems, bones, and cardiovascular and endocrine systems. This condition is caused by inherited or de novo mutations of the NF1 gene at the 17q11.2 chromosomal region, a gene that codes for the protein neurofibromin [7]. It is a relatively common genetic disorder affecting about 1 in 3000 individuals. The features of the disorder are quite diverse and can include dermatologic, cognitive, and osseous diseases [8, 9]. Known focal osseous complications include long bone dysplasia with pseudarthrosis, sphenoid wing dysplasia, nonossifying fibromas of long bones, chest wall deformities (pectus excavatum), and focal, short-segmented scoliosis. The coexistence of hydrocephalus and NF1 is known [10], but the presence of a spinal pseudomeningocele is very rare. The pathogenesis of the pseudomeningocele is a tear of the dural sac with a valve mechanism and the local alteration of the cerebrospinal fluid flow may cause the growth of the defect. When discovered, immediate surgical repair has been recommended especially for large pseudomeningoceles with the goal to prevent fistulous tract and infections [11]. Various treatment options like close observation for spontaneous resolution of small lesions, epidural blood patch, lumbar subarachnoid drainage and synthetic dural patch have been described in the literature for management of pseudomeningoceles with good results [12–14]. It is well known that the presence of dystrophic features in patients with type 1 neurofibromatosis increases the risk of relapse of pseudomeningocele after surgery [15]. The presence of a spinal scoliotic curve with imbalance and the remodeling of foramina and bony structures caused by CSF pulsation could be the possible reason of the multiple relapses of pseudomeningocele in our patient. For these reasons the traditional fat graft and the fibrin glue were not appropriate to repair the dural defect. Today tissue engineering is an emerging multidisciplinary field in which researchers are striving to replace or regenerate damaged or lost tissues. The principle common to tissue engineering is to produce new tissues by seeding cells onto a suitable three-dimensional scaffold in an appropriate growth environment. According to this principle, acellular dermal matrix can be regarded as a natural acellular scaffold to facilitate healing. In this patient the repair of the defect was possible only with an intra-abdominal approach and the use of an acellular dermal matrix (ADM) sutured directly on the dural defect [14]. Acellular dermal graft is processed from banked cadaver skin. The cellular elements are removed in the processing of the allograft but the native collagen and elastin matrix and the basement membrane complex (BMC) are preserved. It is also treated with agents that prevent any viral transmission when implanted. Because the tissue is acellular, it does not produce an antigenic inflammatory response after implantation. This tissue becomes vascularized like autologous tissue after implantation and is used especially in the reconstruction of contaminated abdominal wall defects. In our patient the integration of the implant with the dural sac allowed the reconstruction of the dural defect and the healing of the giant pseudomeningocele. In conclusion, in our particular case ADM was found to be a safe and effective alternative to traditional fat graft for a very big dural wall reconstruction, even in the setting of contaminated fields where also posterior fusion is now allowed [16].
